# Case Report: Deep intronic *PHEX* variant causing aberrant splicing identified by whole genome and targeted RNA sequencing in X-linked hypophosphatemia

**DOI:** 10.3389/fendo.2026.1847219

**Published:** 2026-07-09

**Authors:** Susanne Spranger, Helene Faust, Patricia Duffek, Susanna Schubert, Heyko Skladny, Peter Kovacs, Anke Tönjes, Denny Popp

**Affiliations:** 1 Medical Department III, Endocrinology, Nephrology, Rheumatology, University of Leipzig Medical Center, Leipzig, Germany; 2 Institute of Human Genetics, University of Leipzig Medical Center, Leipzig, Germany; 3 Institute of Human Genetics, University Hospital Schleswig-Holstein, Kiel, Germany; 4 SYNLAB MVZ Humangenetik Mannheim GmbH, Mannheim, Germany; 5 LeiCeM - Leipzig Center of Metabolism, Leipzig University, Leipzig, Germany

**Keywords:** Aberrant splicing, burosumab, case report, deep intronic variant, PHEX gene, RNA sequencing, whole genome sequencing, X-linked hypophosphatemia

## Abstract

X-linked hypophosphatemia (XLH) is a rare, genetically determined disorder of phosphate metabolism, most commonly caused by mutations in the *PHEX* gene. These mutations lead to overexpression of the phosphaturic hormone FGF23, resulting in renal phosphate wasting and impaired bone mineralization. In up to 16% of clinically diagnosed cases, no causative variant can be identified using standard sequencing approaches. We report on a female patient with a clearly defined clinical XLH phenotype, in whom no causative mutation had been detected over several years despite extensive genetic testing. The aim was to identify a previously undetected genetic cause using extended DNA and RNA methods. After unremarkable short-read whole exome sequencing (WES), short-read whole genome sequencing (WGS) was performed. For confirmation of splice effect, RNA was extracted from peripheral blood, amplified via RT-PCR, and analyzed using Nanopore long-read sequencing. A novel deep intronic variant in the *PHEX* gene (c.2070 + 601C>T) was identified and confirmed as *de novo*. The variant caused two aberrant transcripts with pseudoexon inclusions, each leading to a premature stop codon. This aberrant splicing supports the pathogenicity of the variant in the context of a loss-of-function mechanism. Following molecular diagnosis, the patient was successfully initiated on Burosumab therapy, resulting in clinical improvement. This case highlights the diagnostic value of comprehensive genomic analysis and subsequent RNA sequencing for identifying and analyzing deep intronic variants in genetically unexplained cases of XLH. The findings expand the known *PHEX* mutation spectrum and emphasize the importance of re-evaluating patients with a strong clinical diagnosis but previously negative genetic results. In the future, such technologies may play a crucial role in improving diagnostics for rare monogenic diseases.

## 1 Introduction

X-linked hypophosphatemia (XLH) is a rare genetic disorder characterized by defective phosphate homeostasis, resulting in hypophosphatemia, impaired bone mineralization, and progressive skeletal deformities (1). The condition is primarily caused by pathogenic variants in the *PHEX* (phosphate regulating endopeptidase X-linked) gene, which encodes an endopeptidase that regulates phosphate metabolism. These variants lead to the excessive production of fibroblast growth factor 23 (FGF23), a hormone that inhibits renal phosphate reabsorption and suppresses the activation of vitamin D, thus contributing to the clinical features of XLH. Although *PHEX* expresses a protease, it mainly affects the expression of fibroblast factor 23 (FGF23), rather than promoting the degradation of FGF23 (2). The regulatory mechanism between *PHEX* and FGF23 is not completely clear, but it was shown that *PHEX* is a direct transcriptional inhibitor of FGF23 and affects the expression of FGF23 (3).

The diagnosis of XLH is typically confirmed through a combination of clinical assessment, biochemical testing, and genetic analysis. Genetic testing for variants in the *PHEX* gene can confirm the diagnosis but is not absolutely necessary for diagnosis and therapy. The treatment of XLH involves phosphate supplementation and active vitamin D to correct hypophosphatemia and improve bone mineralization. In recent years, a specific therapy has also been developed that is now approved for children and adults. Burosumab, an FGF23-targeting monoclonal antibody, may be used to reduce FGF23 levels and enhance phosphate reabsorption.

Despite the identification of numerous genetic variants in *PHEX*, the genetic landscape of XLH remains incompletely understood, with some patients exhibiting phenotypes that are not fully explained by known variants (4, 5). Recent advances in genomic sequencing technologies have enabled the discovery of rare or previously unidentified variants, providing valuable insights into the genetic etiology of the disorder.

In this report, we describe a novel genetic variant identified in a patient with XLH. This variant has not been previously reported in the literature and was not detected by multiple genetic tests over the last 20 years. This offers new insights into the molecular mechanisms of pathogenic variants in *PHEX* underlying XLH and highlights the importance of renewed genetic diagnosis in patients with a clear clinical diagnosis and a previously undetected genetic cause.

## 2 Methods

### 2.1 Ethics and consent

This study adheres to the principles of the Declaration of Helsinki. This study was approved by the Ethical Committee of the Medical Faculty of the Leipzig University. The authors received and archived written consent from the presented individuals for both study participation and publication of genetic and clinical data.

### 2.2 DNA extraction

Genomic DNA (gDNA) was extracted from EDTA whole blood using the MagCore^®^ HF 16 Plus II Nucleic Acid Extractor. Starting from 400 µl EDTA blood samples, 100 µl DNA elution volume was obtained using the Genomic DNA Whole Blood Kit and 101 cartridge (RBC Bioscience^®^, New Taipei City, Taiwan) according to the manufacturer’s instructions. DNA concentrations and purity (260/280 ratio) were measured with a Tecan Infinite^®^ 200 Microplate Reader (Tecan, Männedorf, Switzerland) using the absorbance and fluorescence modes.

### 2.3 Exome and whole genome sequencing

DNA was enriched for exome sequencing using the TWIST Exome 2.0 Kit (TWIST Bioscience, San Francisco, CA, USA). The library was sequenced with 150 base pairs (bp) paired-end reads on a NovaSeq 6000 device using an S4 Reagent Kit (Illumina, Inc., San Diego, CA, USA). On average coverage of targeted genomic regions was 147× with 99.7% covered at least 10×. Raw data was processed using varfeed followed by a tertiary analysis with the browser-based genomics software Varvis (Limbus Medical Technologies GmbH, Rostock, Germany).

For whole genome sequencing (WGS), library preparation was done using the Twist Library Preparation EF Kit1, 2.0, and Twist Universal Adapter System—TruSeq Compatible, 96 Samples Plate A–D strictly following the manufacturer’s instructions and SOPs. Paired-end next-generation sequencing (2× 150 bp) was then performed on a NovaSeq 6000 device using an S4 Reagent Kit (Illumina, Inc., San Diego, CA, USA). Raw data analysis was performed using Dragen 4.0 implemented in the Emedgene software, and variants were scored and prioritized using the Emedgene software (Illumina, Inc., San Diego, CA, USA). We prioritized all potentially relevant variants in terms of pathogenicity and clinical relevance according to all possible modes of inheritance. We performed a genome-wide detection of CNV/SVs with DragenCNV4.0 and Dragen-Manta4.0 implemented in Emedgene.

The clinically relevant variants were classified according to the ACMG criteria and latest recommendations (6) and the ACGS Best Practice Guidelines for Variant Classification in Rare Disease 2024 (7). The identified variant in *PHEX* was submitted to ClinVar (ID: 3359149) and to *PHEX* LSDB (#0001067986).

### 2.4 Sanger sequencing and multiplex ligation–dependent probe amplification

Bidirectional Sanger sequencing for confirmation and segregation analysis of the parents was performed on the Applied Biosystems 3500 Genetic Analyzer (Thermo Fisher Scientific Inc., Waltham, Massachusetts, U.S.), using primers binding to regions in intron 20 of the *PHEX* gene (forward primer sequence: TTATGCCACCACTCTCAGCC, reverse primer sequence: GCAGGCAAGCAAGAATCCTG). Multiplex ligation–dependent probe amplification (MLPA) analysis of the duplication on chromosome 22 was performed using the P245 Microdeletion Syndromes-1A kit (MRC-Holland, Amsterdam, The Netherlands) on an Applied Biosystems 3500 Genetic Analyzer (Thermo Fisher Scientific, Darmstadt, Germany). Sanger sequences and MLPA were analyzed using the Sequence Pilot Software (JSI medical systems, Ettenheim, Germany) and Integrated Genomics Viewer (IGV), respectively.

### 2.5 X-inactivation

X-inactivation was assessed by methylation-specific restriction digestion (HhaI and HpaII) of genomic DNA and the subsequent amplification of the HUMARA locus (exon 1 of the AR gene). X-inactivation was calculated as the ratio of methylated to non-methylated alleles.

### 2.6 RNA extraction

The purification of intracellular RNA from peripheral blood was performed using the PAXgene Blood RNA Kit (PreAnalytiX, Hombrechtikon, Switzerland) according to of the manufacturers’ instructions. During the isolation, an integrated DNase treatment was conducted to remove gDNA. RNA concentration and purity (260/280 ratio) were measured using a NanoDrop 2000 (Thermo Fisher Scientific, Darmstadt, Germany).

### 2.7 Nanopore sequencing

RNA was reverse transcribed using PrimeScript RT Master Mix (TaKaRa Bio, San Jose, USA), and subsequently parts of the *PHEX* gene (exon 18 to 22) were amplified (forward primer sequence: ACCTGGATCCTTGGTGGTCT and reverse primer sequence GGCAGCTTCTGGTCTGTAGG). A library for long-read sequencing was prepared from the PCR product using the NEBNext Companion Module (New England Biolabs, Frankfurt am Main, Germany) and the 24 barcoding library prep kit SQK-NBD114.24 according to the standard protocol of Oxford Nanopore Technology (ONT, Oxford, UK) with the following adjustments. The incubation time of the end-prep reaction was increased to 30 min at 20°C and 30 min at 65°C. The incubation time of the adapter ligation reaction was adjusted to 60 min at room temperature. The final library was loaded onto a MinION flow cell type R10.4.1 and sequenced on a MinION device Mk1b (ONT). Raw data (fast5 files) were base-called and de-multiplexed using dorado 0.6.2 (ONT). Base-called reads were then aligned against the human reference genome hg38 using splice-aware minimap2 v2.17-r94115. Reads were then inspected manually for potential alternative splicing events using the IGV. In addition to the patient’s sample, three control samples from healthy individuals were processed and sequenced in the same manner.

## 3 Case presentation

A 20-year-old female patient presents with bilateral genua vara and significantly reduced phosphate levels of 0.56 mmol/l. The initial clinical diagnosis of a phosphate-wasting disorder was made at the age of six due to growth disorders, delayed motor development, progressive genua vara, and significantly decreased fasting phosphate levels. At the age of 12 years, a temporary ventrolateral hemiepiphysiodesis was performed bilaterally, followed by a valgus tibial head osteotomy at the age of 16 years.

In addition to the markedly reduced phosphate levels, the patient had a normal calcium level of 2.23 mmol/L and a mild 25-vitamin D deficiency, with a 25-vitamin D level of 26.2 ng/ml, which normalized after supplementation with 1000 IU/day. Bone-specific alkaline phosphatase, as an indicator of osteomalacia, was elevated at 29.3 µg/ml. ß-crosslaps, as a resorption marker, were within the reference range for premenopausal women at 393 pg/ml. Osteocalcin was within the reference range at 25.3 ng/ml, and calcitriol was in the lower reference range at 32.8 pg/ml. The TmP/GFR was significantly reduced at 5.5 mmol/L, indicating markedly decreased phosphate reabsorption in the urine. FGF23 was markedly elevated at 162 RU/ml, explaining the reduced phosphate reabsorption. Parathyroid hormone was within the normal range at 3.86 pmol/L, thereby ruling out secondary hyperparathyroidism.

During childhood, conservative therapy was initiated with phosphate supplementation and active vitamin D following the clinical diagnosis. After turning 18 years, the patient had no further medical follow-ups and thus took phosphate and active vitamin D only irregularly.

The patient exhibits disproportionate short stature with a height of 148 cm. Additionally, she has known sensorineural hearing loss requiring hearing aids and dental caries with multiple tooth extractions.

After transitioning into our outpatient clinic, the patient was referred to the Institute of Human Genetics for genetic confirmation of the disease. Initially, whole exome sequencing (WES), including the *PHEX* gene, did not identify a genetic cause. However, WES analysis revealed a 2.5 Mbp interstitial, heterozygous duplication on chromosome 22: seq[GRCh38] 22q11.21(18173893x2,18906325_21416074x3,21361083x2)dup which is not associated with hypophosphatemia. This duplication might cause short stature and hearing impairment. However, this variant does not explain the hypophosphatemia and was found to be maternally inherited by segregation analysis using MLPA. Moreover, the penetrance was estimated to be about 22% (8). Hence, we did not consider this duplication as s conclusive genetic cause for the symptoms of the proband.

**Figure 1:**
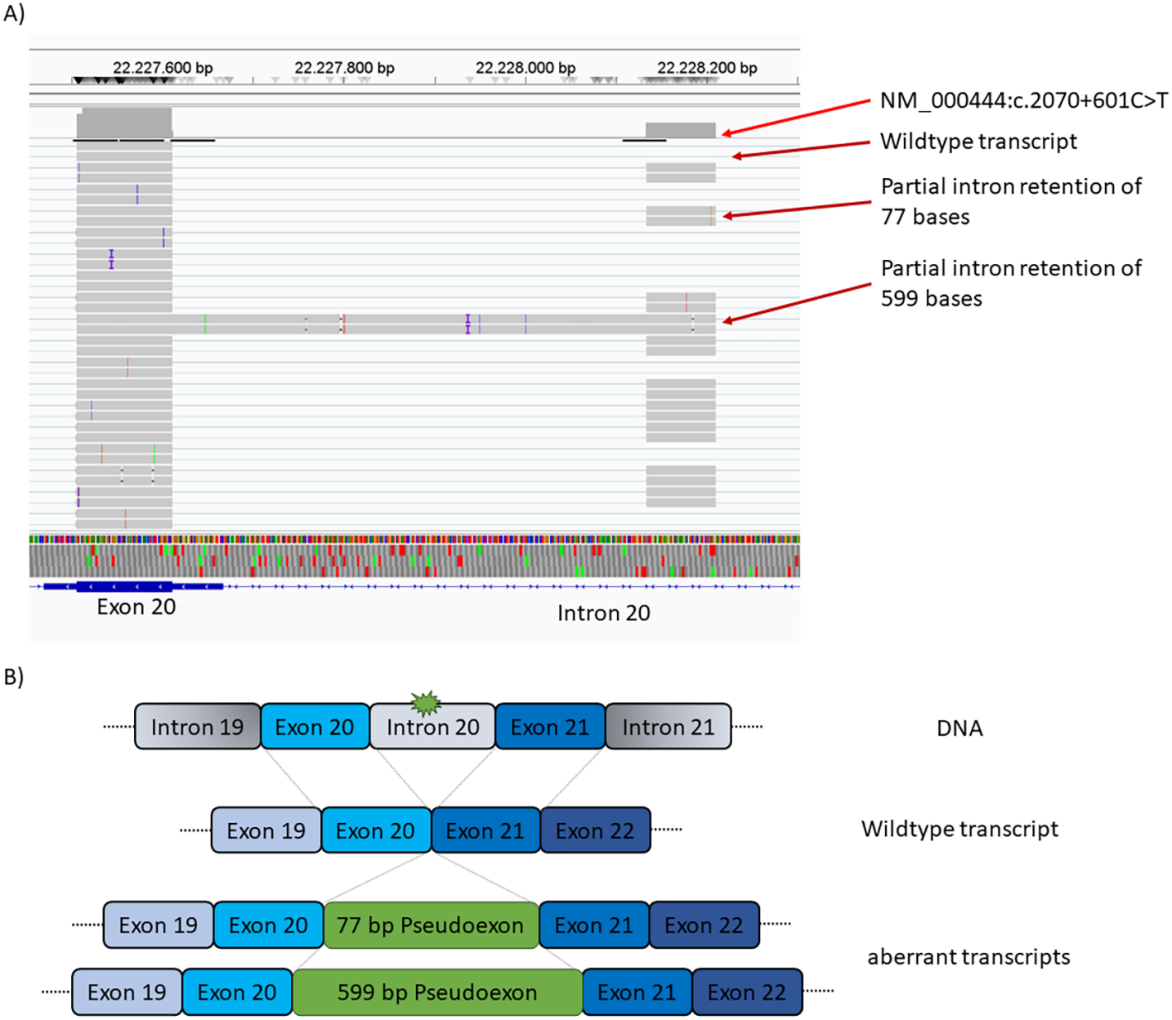
**(A)** IGV screenshot of splice-aware aligned reads of RNA analysis showing the wildtype transcript along with two aberrant transcripts with a partial intron retention of either 77 or 599 bases of intron 20 of the *PHEX* gene. Both aberrant transcripts most likely lead to a frameshift variant. **(B)** Schematic representation of parts of the PHEX gene on DNA level indicating the variant in intron 20 as well as on RNA level showing the wildtype transcript and the two aberrant transcript each including a pseudoexon.

Due to a strong suspicion of a genetic cause of the hypophosphatemia, diagnostics were extended to WGS. WGS analysis revealed a deep intronic variant in intron 20 of the *PHEX* gene: chrX:22228212C>T, NM_000444.6:c.2070 + 601C>T (hg38). This variant is not found in the general population database gnomAD v4 (9). Segregation by Sanger sequencing revealed this variant to be *de novo* as both parents do not have it. An acceptor gain of 78 bp upstream and a donor gain of 2 bp upstream of this variant was predicted in silico (spliceAI) (10). This prediction indicated a potential pseudoexon inclusion between exon 20 and 21. Based on the results of WGS and the *in-silico* prediction, further analysis was focused on *PHEX* transcripts. RNA analysis based on a blood sample was performed to study the impact of the variant c.2070 + 601C>T in *PHEX* on splicing. Next to the wild-type transcript, two aberrant transcripts were identified; see **Figure 1**. The variant causes a partial intron retention of either 77 or 599 bases of intron 20 into the mature mRNA (r.[2070_2071ins2070 + 523_2070 + 599,2070_2071ins2070 + 1_2070 + 599] with 49% and 3% of the obtained reads, respectively). Both aberrant transcripts most likely lead to a frameshift variant (p.([His690_Val691ins*14, His690_Val691ins*11])), which is in line with the described pathomechanism for a *PHEX*-associated XLH (4). Based on the collective evidence, this variant was classified as pathogenic [PVS1(RNA), PS2_MOD, PM2, and PP4 criteria (ACMG 6)].

The analysis of the X-inactivation gave conflicting results as the two applied restriction enzymes resulted in a different amplification pattern. Restriction with HhaI gave hints for a skewed X-inactivation. However, this could not be confirmed by restriction with HpaII. Hence, results of X-inactivation remained inconclusive.

After identification of a pathogenic deep intronic *PHEX* variant, therapy with Burosumab was initiated following appropriate patient education and confirmation of the indication. The initial dosage was 1 mg/kg body weight, corresponding to 60 mg subcutaneously once per month. Under this treatment, rising phosphate levels were already observed (trough level 0.63 mmol/L, peak level 0.76 mmol/L); however, these were still not within the target range, making a gradual dose increase up to 70 mg Burosumab necessary. With this adjustment, a phosphate trough level of 0.81 mmol/l, just below the reference range, was achieved. The TmP/GFR showed a marked increase to 33 mmol/l. Bone-specific alkaline phosphatase showed a marked decrease and, at 13.7 µg/ml, was within the normal range, demonstrating the regression of osteomalacia. Clinically, 6–9 months after initiation of Burosumab therapy, the patient showed a reduction in bone pain, and the need for analgesics decreased.

## 4 Discussion

X-linked hypophosphatemia (XLH) is primarily caused by mutations in the *PHEX* (phosphate regulating endopeptidase X-linked) gene. However, in approximately 16% of clinically diagnosed patients, standard genetic testing (such as Sanger sequencing, MLPA, or exome sequencing) does not reveal a causative mutation (11). Our present findings contribute to closing this diagnostic gap by demonstrating how a deep intronic variant, which was undetectable by earlier sequencing methods, can be pathogenic through aberrant splicing.

In this study, we describe an adult female with typical symptoms of XLH, which was confirmed by clinical and biochemical findings. However, a genetic cause could not be identified by WES, as no clinically relevant variant in the *PHEX* gene or another gene related to hypophosphatemia was detected. In contrast, by WGS, a deep intronic pathogenic *de-novo* variant in *PHEX* was detected. Evidence for its pathogenicity was gathered by segregation analysis using Sanger sequencing and transcript analysis using Nanopore sequencing. By the latter, two pseudoexon inclusions were confirmed. This results in two aberrant transcripts, both introducing premature stop codons. The presence of two different aberrant splicing outcomes may reflect alternative cryptic splice site usage triggered by the single nucleotide change. This is in line with the established *PHEX* disease mechanism (12, 13), where loss-of-function mutations typically result in the overexpression of FGF23 and subsequent phosphate wasting. Hitherto, 1047 unique variants (as accessed on the 5th of May, 2026) in *PHEX* have been described (*PHEX* LSDB), with the majority resulting in a truncated PHEX protein (13). The herein identified variant was not described yet in any other publication or the relevant databases (ClinVar, HGMD, and *PHEX* LSDB).

Besides this deep intronic variant, two other deep intronic variants in *PHEX* (c.2147 + 1197A>G in intron 21 and c.849 + 1268G>T in intron 7, respectively) have been reported (14–16). Both variants were shown to cause an inclusion of pseudoexons, which would result in a likely loss of function as well. Hence, to the best of our knowledge, this study presents a third deep intronic variant in *PHEX* causing XLH.

Even though X-inactivation is commonly found to be skewed in the case of an X-chromosomal disease (17), the X-inactivation pattern in XLH patients was found to be random and hence similar to the general population (18, 19). According to literature reports, selective inactivation of the allele carrying a pathogenic variant can result in a normal phenotype (18, 20). Hence, random X-inactivation would be expected for XLH patients. In our case, X-inactivation yielded inconclusive results, illustrating the technical limitations and tissue specificity of such assays. Given that X-inactivation can vary between tissues, peripheral blood may not always reflect the inactivation status, for example, in bone, one of the primary organs affected in XLH.

Recently, 831 unrelated individuals with clinical XLH or suspected genetic hypophosphatemia underwent genetic testing, applying 13-gene panel sequencing (11). While most affected individuals had a (likely) pathogenic variant or variant of uncertain significance in the *PHEX* gene (69%) or another gene related to hypophosphatemia (15%), 135 individuals (16%) remained without a genetic diagnosis. This diagnostic gap might be indeed explained by deep intronic variants, especially in the *PHEX* gene, which were missed by panel sequencing. Furthermore, our case raises the question of diagnostic strategy. In case of a clinical suspicion of XLH and negative WES as a state-of-the-art genetic diagnostic approach, it is worthwhile to follow up with WGS or even whole transcriptome sequencing to identify deep intronic variants as shown here and recently in the literature (4, 16). However, its higher cost and complexity limit the routine application of WGS. Alternatively, targeted whole gene sequencing may serve as a cost-effective middle ground in future diagnostics as proposed already for other entities (21). Such a panel would target the whole *PHEX* gene, including non-coding regions of *PHEX* and related phosphate-wasting genes. However, as sequencing costs continue to decline, WGS might become increasingly accessible, providing comprehensive coverage in a single step.

Identification of deep intronic variants should be complemented by transcript analysis to analyze and confirm their predicted effects. A targeted analysis is usually performed using RT-PCR and subsequent Sanger sequencing (4) and can be based on a blood sample. Even though the expression in blood is rather low (0.03 transcripts per million according to the GTEx Portal), we could successfully demonstrate splicing effects in the *PHEX* gene. Here, a single genomic variant caused two different aberrant transcripts, which could be readily detected as we applied RT-PCR combined with Nanopore sequencing. Using the conventional approach based on RT-PCR and Sanger sequencing, the identification of multiple splicing effects would not be possible, and minor effects might remain undetected. Hence, next-generation sequencing approaches like Nanopore sequencing are better suited for RNA analysis to reliably detect several different splicing effects occurring at once and also minor effects.

This case report highlights the diagnostic value of advanced genomic tools, particularly long-read nanopore sequencing, in deciphering cryptic genetic variants in patients with XLH, where conventional methods fail. It demonstrates the clinical relevance of continuous improvement of modern and increasingly cost-efficient methods of genetic testing and the meaningfulness of re-examining patients. Especially in cases with a clear monogenic cause, this allows for more accurate diagnoses and helps to identify diseases more precisely and potentially improve treatment.

## 5 Conclusion

In conclusion, this case underscores the importance of re-evaluating patients with a strong clinical diagnosis of XLH but negative genetic findings using newer and more comprehensive technologies. The identification of a pathogenic deep intronic *PHEX* variant expands the mutational spectrum of XLH and exemplifies how integrating advanced sequencing with targeted RNA analysis can elucidate pathogenic mechanisms previously missed. Moving forward, the implementation of long-read transcriptome analysis may become a crucial tool in the diagnostic pipeline for rare genetic disorders, especially those with elusive or non-coding variants.

## Data Availability

The original contributions presented in the study are included in the article. Further inquiries can be directed to the corresponding author.
